# Children’s Learning from Touch Screens: A Dual Representation Perspective

**DOI:** 10.3389/fpsyg.2016.01220

**Published:** 2016-08-12

**Authors:** Kelly J. Sheehan, David H. Uttal

**Affiliations:** Department of Psychology, Northwestern University, EvanstonIL, USA

**Keywords:** touch screen, symbol, symbolic medium, dual representation, tablets, symbolic understanding

## Abstract

Parents and educators often expect that children will learn from touch screen devices, such as during joint e-book reading. Therefore an essential question is whether young children understand that the touch screen can be a symbolic medium – that entities represented on the touch screen can refer to entities in the real world. Research on symbolic development suggests that symbolic understanding requires that children develop dual representational abilities, meaning children need to appreciate that a symbol is an object in itself (i.e., picture of a dog) while also being a representation of something else (i.e., the real dog). Drawing on classic research on symbols and new research on children’s learning from touch screens, we offer the perspective that children’s ability to learn from the touch screen as a symbolic medium depends on the effect of interactivity on children’s developing dual representational abilities. Although previous research on dual representation suggests the interactive nature of the touch screen might make it difficult for young children to use as a symbolic medium, the unique interactive affordances may help alleviate this difficulty. More research needs to investigate how the interactivity of the touch screen affects children’s ability to connect the symbols on the screen to the real world. Given the interactive nature of the touch screen, researchers and educators should consider both the affordances of the touch screen as well as young children’s cognitive abilities when assessing whether young children can learn from it as a symbolic medium.

## Introduction

Since the introduction of touch screen technology, new media platforms such as tablet computers and other handheld devices have been marketed to and widely used by children of young ages (Common Sense Media, 2013). Compared to other technologies used by children, touch screen devices are unique because children can use them in many ways, such as for watching videos, e-book reading, Skyping with grandparents, and more. For many of its uses, such as e-book reading and watching videos, learning from the touch screen requires that children appreciate the symbolic nature of the touch screen. Children can learn by connecting the entities depicted on the screen with their referents in the real world. But does the child understand that the animals they learned about in the e-book represent animals in the real world? Can the child connect concepts learned from a video on a touch screen to her everyday experiences?

For traditional symbols, such as pictures and text, making the leap from symbol to referent requires that children develop *dual representation* – they must represent both that the symbol is a concrete object while also representing that the symbol refers to something other than itself (DeLoache, 1989; DeLoache et al., 1997). However, the touch screen is not a traditional symbolic medium. It is unique in that it is interactive; children can directly manipulate the screen, which responds instantly to their touch. However, being able to manipulate the screen does not necessarily mean children can learn from it. We review traditional symbolic research that suggests manipulating the touch screen may lead children – specifically toddlers and preschool-aged children – to focus on the screen itself rather than on what the entities on the screen represent (DeLoache, 2000). But in contrast, we also review more recent research that suggests the interactivity may help children connect entities on the screen to their referents, perhaps allowing them to circumvent the potential difficulty caused by dual representation.

The purpose of this paper is to consider both the potential negative and positive effects of touch screen interactivity on children’s ability to understand the symbolic nature of entities represented on the screen, but it is important to note that the potential effects likely depend on children’s age and the touch screen activity. For example, in this paper, we discuss the possibility that interactivity may hinder preschool-aged children’s learning from a symbol they typically learn from without interactivity, but may promote learning for toddlers who typically struggle to learn from that symbol. Additionally, the touch screen can be used for many different symbolic activities, some of which are interactive (e.g., reading an interactive e-book) and some of which are not (e.g., watching a video). In this paper, we take the perspective that interactivity alters how children view the touch screen as a symbolic medium, and therefore affects their symbolic learning from both interactive and non-interactive activities. However, it is possible that children learn differently from interactive versus non-interactive activities. While this perspective will not expand on all these possibilities, we do discuss the general potential effects of interactivity, age, and touch screen activity on children’s symbolic transfer from the touch screen.

## The Effect of Interactivity on Dual Representation

It is often difficult for young children to “see through” a symbol to the referent that it represents (DeLoache, 2000). Instead children often focus on the symbol itself rather than on the entity it refers to. For example, 9-month-old infants will physically manipulate a picture, treating the picture like the object it represents rather than appreciating it as merely a representation (DeLoache et al., 1998; Pierroutsakos and DeLoache, 2003). Similarly, when asked to use a scale model to find a hidden object in a larger room, 2.5-year-olds fail to use the model as a representation but succeed in finding the object when they are made to believe that the model magically grew to be the room (DeLoache, 1987; DeLoache et al., 1997). In both of these cases, children’s symbolic failure stems from a lack of dual representation; they focus on the symbol as an object in itself rather than on it being a representation for something else.

Considering children’s difficulty with dual representation, emphasizing a symbol’s status as an object or entity can hinder children’s understanding and use of that symbol, while de-emphasizing its status as an object can promote children’s symbolic use. For example, DeLoache (2000) found that when children were asked to use a scale model as a symbol for a room, 2.5-year-olds’ performance was facilitated when the model was put behind glass, which prevented them from playing with model and therefore helped them view the model as a representation and not as a toy. In addition, 20-month-old infants learned fewer novel labels for three-dimensional pictures in a pop-up book compared to two-dimensional pictures in a traditional picture book (Tare et al., 2010). Here, making the pictures three-dimensional – and therefore objects – hindered toddlers’ symbolic learning. When children view a symbol as an appealing object, it is more difficult for them to represent both that object and the referent that it represents. Symbols that are salient physical entities are more difficult for children who are developing dual representational abilities to understand.

However, it is not just concrete objects that can hinder young children’s dual representation of symbols; two-dimensional screens can also pose a dual representation problem for young children. Many researchers have suggested that children’s difficulty with the dual representational nature of the screen is one reason why young children often struggle to learn from video, a phenomenon that has been termed the *video deficit effect* (Anderson and Pempek, 2005; see also Barr, 2010; Krcmar, 2010). For example, 24-month-old infants struggle to use a video of an object being hidden in a room to find the object, but succeed in using the video when they are made to believe they are directly seeing the toy being hidden in real life (Troseth and DeLoache, 1998; Schmitt and Anderson, 2002). Much other research shows that young children are relatively poor at learning information presented on a television compared to learning from a face-to-face interaction with a person, such as imitating an action sequence (Barr and Hayne, 1999) or learning new words (DeLoache et al., 2010). To young children, the image on the screen is just an image. They may not realize that the image can inform them about objects and actions in their lives. Therefore young children need to learn to appreciate that a video image is not just something on television, but also potentially represents something real.

The cost of appealing symbols, as well as children’s difficulty learning from screens, suggests that the interactive affordance of touch screens may pose a symbolic impediment for young children because it may lead children to focus on the screen that they are manipulating rather than on what the image on the screen stands for. Children interact with the touch screen in a way that may lead them to conceptualize the screen as being an appealing object. Therefore, because the touch screen is designed to be manipulated, young children’s interaction with it may lead them to focus on the touch screen itself rather than on what the images on the screen represent. Interactivity may emphasize that the screen is an object in its own right rather than as a medium for representing objects.

Touch screens may pose a problem for dual representation not only because of their interactive features, but also because they are multimodal and are often used for playing games, which may lead children to conceptualize the device as being a toy. Toys are especially appealing objects, which means it is very difficult for children to see through toy-like symbols to the referents they represent. For example, research shows that when children are asked to use a toy such as a doll as a symbol, 2.5-year olds perform poorly when asked to map between the doll and their own body (DeLoache and Marzolf, 1995; Herold and Akhtar, 2014). Strong evidence for the disadvantage of toy-like symbols come from a study in which 3-year-olds played with a scale model like a toy for 10 min before using it as a symbol to find a hidden object in a larger room (DeLoache, 2000). While 3-year-olds typically found the hidden object on 75 percent of their searches, playing with the model beforehand led children to find the hidden object on only 44 percent of their searches. In the same way that 3-year-olds’ use of a scale model as a toy hindered their understanding of it as a symbol, young children’s use of a touch screen device as a toy may hinder their later understanding of the screen as a symbolic medium.

If children use touch screens as toys by playing games on them, they may form expectations about the devices as being a form of entertainment rather than a tool for learning. Children’s expectation about a video has been shown to affect their ability to learn from it as a symbol. For example, research shows that children imitated less from a video viewed on their own television (that they usually used for entertainment) compared to an unfamiliar video monitor in a laboratory (Strouse and Troseth, 2008). Children’s previous (and possibly more frequent) experiences using a two-dimensional screen as an appealing source of entertainment may hinder their later ability to use it as a symbol. Therefore, children’s interaction with touch screens, their conceptualization of them as toys, and the subsequent expectations they form about the purpose of touch screens are all possible reasons why children may struggle to learn from symbols represented on the touch screen.

## Can Interactivity Alleviate the Need for Dual Representation?

Despite the implications of research on traditional symbols and symbolic media, there are reasons to believe that the interactivity of the touch screen may not hinder children’s understanding of it as a symbolic medium, but rather may promote children’s learning from the symbols represented on the screen. As mentioned above, the touch screen is a unique symbolic medium, and is almost entirely different from other symbolic media because it immediately responds to the child’s touch. Although, we can draw upon research on traditional symbolic media to make inferences about the possible effects of interactivity on children’s symbolic understanding, the touch screen’s interactivity may set it entirely apart, meaning the results of previous research may not generalize to it. The touch screen may be in an entirely unique symbolic class of its own.

In this section, we consider the potential positive effects interactivity may have on children’s ability to learn from the touch screen. It is possible that the interactive nature of the touch screen can actually promote children’s symbolic use of it because the interactivity links the screen with the child’s experiences in the real world. From this perspective, the interactive aspect of the touch screen does not create an impediment for dual representation, but actually reduces or circumvents the need for dual representation. Research shows that children learn better from characters or people on a screen when they are *socially contingent* to the child – or in other words, when they are responsive to a child’s actions or vocalizations (Troseth et al., 2006; Krcmar, 2010; Roseberry et al., 2014). Contingency is important because it helps the child realize that the person or entity on the screen is relevant to the child, and therefore that the child can learn from that person or entity. The physical contingency of the touch screen may help children learn from it in a similar way: The screen’s immediate response may help children see a symbol as relevant and therefore focus their attention on it – and not other irrelevant entities on the screen. If contingency helps children focus their attention on a particular symbol on the screen, it may help them connect the symbol to its referent and not to other entities that are present.

Importantly there is evidence that interactivity helps children learn from screen media. Lauricella et al. (2010) asked 2.5- to 3-year-olds to participate in a hide-and-seek game in which children either observed an adult finding a hidden object, watched a video revealing where the object was hidden, or played an interactive computer game in which a keyboard response revealed where the object was hidden in the room. When children later searched the room themselves, the 3-year-olds who played the interactive computer game performed just as well as those who observed an adult, and both groups performed significantly better than those who passively watched a video. Although this study did not include a touch screen device, the results suggest that the contingent nature of the game facilitated children’s appreciation of the symbol-referent relation compared to passively watching a video, and that interactivity may be an important means by which young children learn from screen media.

More recent research by Kirkorian et al. (2016) suggests that the contingency of touch screen devices may indeed promote children’s symbolic understanding, but the benefits of interactivity may depend on age. The researchers asked 2-year-olds to watch a video of a person on a touch screen label a novel object, and either had children passively watch, tap anywhere on the screen to hear the label, or tap the location of the object on the screen to hear the label. The researchers found that while tapping the location of the object facilitated word learning for younger 2-year-olds, this manipulation hindered learning for older 2-year-olds who learned the novel word when they passively watched with the video. Choi and Kirkorian (2016) also found a similar effect of contingency and age in an object-retrieval task in which children either passively watched on a touch screen where an object was hidden on a felt board, tapped anywhere on the touch screen, or tapped a specific location on a touch screen to reveal the hiding location. Again, younger 2-year-olds were better at retrieving the object on a corresponding felt board when they tapped a specific location, but older 2-year-olds performed worse when tapping a specific location compared to the other conditions. The researchers suggest that the interactivity benefitted the younger 2-year-olds by guiding their selective attention to target information, but it hindered older 2-year-olds’ performance because the contingency led to over-contextualization: their learning became tied to the context in which the learning took place, which impeded their symbolic transfer.

This research highlights the perspective that touch screens’ interactivity may promote children’s ability to connect objects represented on the screen with their referents, and also suggests the influence of interactivity on symbolic understanding may depend on age and the specific touch screen task. For example, Zack et al. (2009) found that 15- to 16-month-old infants could imitate a novel action performed on an object represented on a touch screen, but struggled to transfer that action to a three-dimensional object (see also Barr, 2010). In comparison, the younger 2-year-olds in Choi and Kirkorian (2016) could transfer from a two-dimensional interactive screen to a three-dimensional apparatus. Depending on the symbolic touch screen activity (e.g., learning new words, learning actions for objects), interactivity may have different effects for different ages.

## Summary and Conclusion

In this paper, we considered the possibility that the interactive nature of the touch screen may affect children’s ability to learn from it as a symbolic medium. First, we adopted a traditional symbolic perspective: interactivity may make the touch screen an appealing object to children, which increases the need for dual representation and therefore may render it a difficult symbolic medium for young children to learn from. For example, research on children’s symbolic understanding of dolls, pop-up picture books, and scale models provide support for the view that emphasizing the toy-like, object status of these symbols hinders children’s ability to learn from them (DeLoache and Marzolf, 1995; DeLoache, 2000; Tare et al., 2010). In the same vein, we suggest that the manipulative, toy-like use of the touch screen may affect the way children conceptualize and form expectations about it. It may be difficult for young children to look past their entertainment value while also appreciating that the entities on the screen can represent real objects or entities, and therefore be used for learning.

However, we also considered the perspective that the very aspect of touch screen devices that may create an impediment for children – the touch screen itself – may also help children connect symbols on the screen to their referents in the world. Touch screens may promote learning by providing a contingent response, which has been shown to help children learn from other symbolic media, such as computers and video, and may help focus children’s attention on the symbol. This possibility is supported by recent research that shows that interacting with a touch screen promotes 2- and 3-year-old children’s ability to connect a symbol on the touch screen to its referent (e.g., Choi and Kirkorian, 2016; Kirkorian et al., 2016). This research also suggests that the effect of interactivity may depend on age; for older children, interactivity may be more distracting than helpful, largely because older children may already be able to transfer from the touch screen during certain touch screen activities without interacting with it.

Nonetheless, it is important to continue pursuing research that is aimed at understanding how the effect of interactivity may change with age and the symbolic touch screen activity (e.g., interactive vs. non-interactive). With more research, educators, parents, and researchers will be better informed of how the unique affordances of the touch screen affect children’s ability to “see through” it as a symbolic medium. Ultimately it can help them assess the value of the touch screen as a symbolic medium, which has implications for its value as a tool for learning at different ages. While the interactive appeal of touch screens may directly impede upon children’s ability to learn from them, it is possible that the interactivity of touch screens may be the very feature that helps children connect symbols on the screen to their referents in the real world.

## Author Contributions

KS contributed to the conception of the work, the intellectual content, the drafting the work, and gave final approval of the version to be published. DU also made substantial contribution to the conception and intellectual content of the work, editing and revising it, and gave final approval of the version to be published. Both KS and DU agree to be accountable for the content of this work.

## Conflict of Interest Statement

The authors declare that the research was conducted in the absence of any commercial or financial relationships that could be construed as a potential conflict of interest.
